# ﻿Ten lessons learned from the mass digitisation of a herbarium collection

**DOI:** 10.3897/phytokeys.244.120112

**Published:** 2024-07-02

**Authors:** Sofie De Smedt, Ann Bogaerts, Niko De Meeter, Mathias Dillen, Henry Engledow, Paul Van Wambeke, Frederik Leliaert, Quentin Groom

**Affiliations:** 1 Meise Botanic Garden, Nieuwelaan 38, 1860 Meise, Belgium Meise Botanic Garden Meise Belgium

**Keywords:** Biodiversity informatics, digitisation, herbarium specimens, natural history collections, taxonomy

## Abstract

Worldwide, herbaria maintain collections of reference specimens representing global plant diversity. These collections are a valuable resource for fundamental botanical research and applied scientific research across various disciplines, and play a significant role in addressing major societal challenges such as biodiversity conservation. The digitisation of herbarium specimens and their online dissemination is one of the most important recent developments in the curation of these collections. Digitisation significantly enhances access to the collections for the research community and facilitates large-scale analysis of biodiversity data. Digitisation also provides a means for preserving the physical specimens, as it reduces the need for handling and transportation. Rapid technological developments have greatly accelerated the rate of databasing and digital imaging of collections. Meise Botanic Garden recently completed a six-year project to mass digitise its herbarium collections of about 3 million specimens mounted on sheets and through this process we have learned valuable lessons. We have captured our experience in 10 recommendations for other collection-holding institutions to take inspiration from as they start planning their own digitisation efforts. We also present case studies where we delve deeper into certain topics as examples.

## ﻿Introduction

As custodians of nomenclatural type specimens, herbaria are the foundation of plant taxonomy. They are also important as source material for new research as well as vouchers for past research. Data from herbaria have been used to assess biodiversity and monitor environmental changes (Vellend et al. 2013; Rawal et al. 2015); for phytogeography (Barney 2006; Lavoie 2013; Groom 2015; Vieira et al. 2021); in invasion biology (Fuentes et al. 2013); the history of science (Groom et al. 2014); building the plant tree of life (Bakker 2017) and many more subjects (Besnard et al. 2018; Carine et al. 2018; Heberling et al. 2019). Specifically, the Meise Botanic Garden herbarium’s contribution to research can be illustrated through the GBIF resources search tool (https://www.gbif.org/resource/search?contentType=literature&gbifDatasetKey=b740eaa0-0679-41dc-acb7-990d562dfa37). These examples represent just the tip of the iceberg of what could be uncovered from the complete digitisation of specimens in herbaria and, in doing so, they fulfil target 21 of the Global Biodiversity Framework: to make data and knowledge on biodiversity globally available (https://www.cbd.int/gbf/targets/21). Furthermore, once specimens are digitised, the images are a target for machine learning to extract yet more data at increased speed and volume (Pearson et al. 2020; Nieva de la Hidalga et al. 2022). Finally, digitisation avoids unnecessary handling of the physical specimens, as this can cause damage or loss. Concurrently, it holds promise in augmenting the demand and utilisation of specimens (both digital and physical) by facilitating increased discovery.

The world’s 3,522 active herbaria collectively house approximately 400 million specimens, providing comprehensive coverage of known plant life and a substantial representation of fungi (Thiers 2022). Together these herbaria are likely to possess at least one specimen of every known plant species. Yet, there is more to be done, the Global Biodiversity Information Facility (GBIF) now links to more than 118 million preserved specimens of Plantae and Fungi, meaning the majority of specimens are still to be imaged, transcribed and disseminated digitally.

Within Europe, the Distributed System of Scientific Collections (DiSSCo) is an emerging research infrastructure that aims to unify access to biodiversity and geodiversity specimens under common standards. Users of European collections will have access to, and be able to use, the full range of specimens and their data from across European institutions. Towards this vision considerable ground work has been done by creating a website with digitisation guides (https://dissco.github.io/) and the DiSSCo Knowledge Base (https://www.dissco.eu/services/knowledge-base/). Additionally, the iDigBio project (https://www.idigbio.org/) in the United States of America has extensive documentation on the digitisation of collections.

The herbarium of Meise Botanic Garden (BR) is, with its collection of around 4 million specimens, the 15^th^ largest herbarium in the world, and is part of the DiSSCo infrastructure. In 2021 the Garden completed two 3-year projects to mass digitise (imaging the specimens and transcribing the label information) its herbarium collections of 2.8 million sheets. During the first project (2015-2018) 1.2 million herbarium sheets from the African and Belgian collection were digitised. During the second project (2018–2021), we have digitised 1.4 million specimens from the general herbarium. Through these two projects we have learned valuable lessons. Digitising a collection is a big undertaking for a herbarium, involving everyone from curators, technicians and scientists to management, human resources, ICT and accounting (Helminger et al. 2020; De Smedt and Bogaerts 2022a, 2022b; Thompson and Birch 2023). The rewards are numerous and it is well worth the effort, but it is important to know what you are letting yourselves in for and plan accordingly. Here we’ve tried to capture our experience in 10 recommendations for other institutions to take inspiration from as they start planning their own digitisation efforts. We also present case studies delving deeper into some of the issues we faced as examples.

## ﻿10 lessons learnt from the mass digitisation of a herbarium collection

### ﻿1. Knowing yourself is the beginning of all wisdom ― Aristotle

Just as it is easy to assume you know yourself before life proves you wrong, it is easy to assume you know your collection. The process of digitisation delves into every dark corner and if there are skeletons hiding, they will be discovered. Most collections do have rough counts of their holdings, but few are really sure how many objects they actually have, because collections are compiled over hundreds of years and not always well documented. A detailed inventory of a representative tenth of your collection can be extrapolated to the entire collection (De Smedt and Bogaerts 2022b, https://dissco.github.io/Digitisation/PreDigCuration/PDCaseStudies.html#estimation-of-the-numbers-of-the-african-and-belgian-herbarium-collection-at-meise-botanic-garden). This will give you a good idea of how many objects you have, so that you can confidently budget for digitising that number (Table 1). While counting, you can also perform other checks: What is the curatorial state of the objects? Where do they come from? What size are they? What information is available on the covers, shelves and cupboards? Are the objects barcoded? How fragile are they? etc. (De Smedt and Bogaerts 2022b; Van Baelen et al. 2022).

**Table 1.** Example of estimated numbers and actual counts of specimens in the African herbarium at Meise Botanic Garden. Estimated numbers are based on a 10% count of the collection.

	African herbarium	DR Congo, Rwanda and Burundi	Other African countries
Estimated number	904,003	515,784	399,218
Actual counts	953,748	520,106	432,642

**Figure 1.**
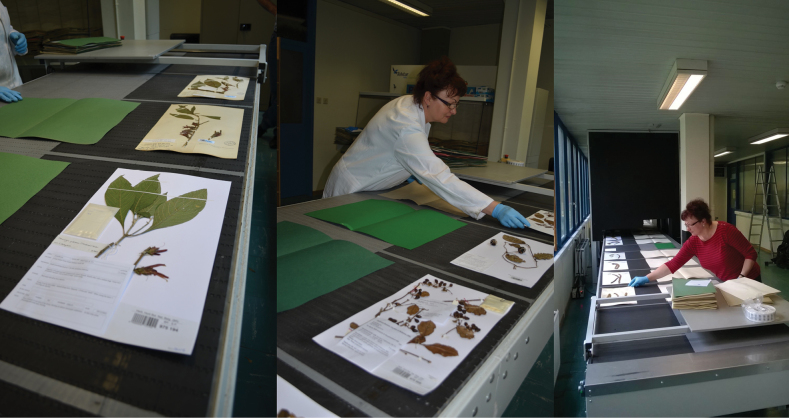
Mass digitisation of herbarium specimens on a conveyor belt at Meise Botanic Garden, allowing the imaging of 3,000–5,000 specimens per day.

Different types of objects require a different approach to digitisation and should be processed together. Some collections are more amenable to mass digitisation than others and require less expertise from digitisers. Flat herbarium sheets, for instance, are much easier to digitise using conveyor belts (Fig. 1) than blocks of wood or specimens in jars or envelopes.

The way specimens are stored can also provide information. If the specimens are arranged by scientific name or country of origin, then during digitisation the name or country only needs to be noted each time it changes in the sequence.

### ﻿2. Prioritise (if lack of money forces you to do so)

Whether you’re digitising your entire collection or just part of it, you have to prioritise the order. Every collection has its own priorities, but you should consider the size, collection type, origin, state of curation, scientific importance, historical importance and stakeholders like internal and external scientists, policy makers and funding bodies (Ahl et al. 2023).

Based on our experience, we recommend digitising the entire collection - in our case all specimens mounted on sheets. It is faster and more cost-effective than to select subcollections or, even worse, select individual specimens. Don’t underestimate the logistics of retrieving and moving specimens around to be digitised. Partly digitising the collection can also create a significant management overhead and can imply different approaches to update the collection.

### ﻿3. Learn from other people’s successes - and mistakes

Do not reinvent the wheel. Learn from others’ experiences and mistakes. Visit other institutes and talk to the project managers who have already gone through the process. Ask for tips and tricks, do’s and don’ts. Use their experience to make your mass digitisation project run as smoothly as possible. If you think you have a better way of doing things, talk it over with someone with experience. Get to know where the pitfalls are, and things you need to take into account when setting up a project. One way to meet the community is through the Consortium of European Taxonomic Facilities (CETAF) who run a specific working group on digitisation. Use guidelines that are already available (https://www.dissco.eu/services/knowledge-base/; https://dissco.github.io/; https://osf.io/eaz38/wiki/home/; https://www.idigbio.org/wiki/index.php/Digitization_Resources) and adapt them to your needs.

Also, talk to digitisation companies that have worked with similar collections to see what is possible with your collection. They may see your collection and project from a different perspective and they can come up with solutions that you may not have thought of. This will also help you write the tender if you are planning to outsource any of the processes. Ensure you familiarise yourself with government and institutional procurement guidelines before you reach out to digitisation companies.

### ﻿4. Decide whether you do it yourself or have it done for you

A mass digitisation project requires many resources, not only money to pay for equipment, supplies and outsourcing, but also personnel with the right skills. You will need people to maintain and prepare the collections, project managers, human resource managers, financial managers, technical and informatics personnel (De Smedt and Bogaerts 2022b; https://dissco.github.io/Digitisation/PreDigCuration/PDCaseStudies.html#staff-list-for-mass-digitisation-project-doe-at-meise-botanic-garden). Match the right profiles to the right tasks to have an overview on the resources that are needed to conduct each task in the process. Some people will have to wear more than one hat and it is important that those hats fit comfortably. An overall project manager dedicated to the digitisation project is an absolute must to make the project successful. Take into account that some daily tasks will move to the background, as more time is going to the digitisation project.

Do you have enough financial resources in your organisation? Or do you need to apply for funding? Explore opportunities regionally, governmentally, internationally, to a foundation or elsewhere. Consult other institutions to find out how they acquired funding for digitisation. Also, be aware that there are strings attached to funding. There may be restrictions on what the money can be used for, some funding agencies only supply money for subcontracting, while others pay for supplies or hiring personnel.

Based on the available funding, that needs to be in place prior to starting, and resources, you can decide which processes to outsource and which to do in house. Possible tasks to outsource may be the restoration, decontamination, mounting, barcoding, packing and transport of specimens, imaging, quality control of the images, long term preservation of the images, transcription of the label data, or website development. Remember that when you are outsourcing, you still need someone in your organisation who will coordinate the relationship with the external partners.

### ﻿5. Make a plan

**Figure 2.**
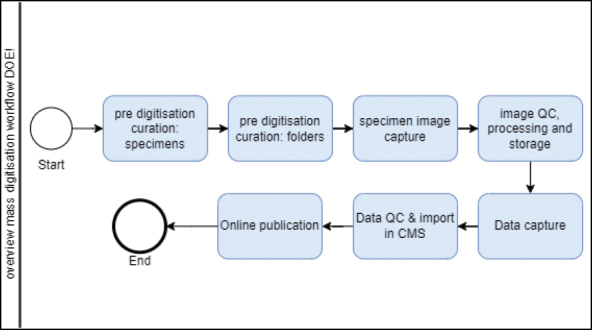
Overview of the mass digitisation workflow for outsourcing at Meise Botanic Garden (based on De Smedt and Bogaerts 2022a).

Now you can use your list of resources to prepare your plan. It should include the necessary workflows (Fig. 2; https://dissco.github.io/HerbariumSheets/MeiseBGHerbariumSheets.html), procedures and tracking systems for all the steps in the digitisation process, whether or not they are outsourced or done in house. Write down what you want and how you want to get it done in as much detail as possible. Don’t forget quality control of the images and the data (see lesson 8), because it is easily underestimated what a huge and important task it is.

When writing tenders for the parts you want to outsource, be as specific as you can to clarify your needs, use examples and add additional information like internal quality control procedures, floor maps, images of your collections, descriptions of your collections, especially where hazardous materials are used. When outsourcing transcription, examples of labels, handwritings and signatures help frame discussions. Clarifying upfront how the tender applications will be evaluated will avoid misunderstandings. Include how well the applicants organise their answers to the tender proposition.

### ﻿6. Go shopping

Once you have your plan ready, you can start listing the necessary supplies. You will definitely need barcodes for each specimen. If you want to replace old folders/jars/boxes, make sure you order enough. It is cheaper to buy in bulk. If you plan to restore specimens before starting the digitisation process, make sure you have adequate supplies and storage space before starting.

The IT infrastructure for image storage, software for image quality control and data transcription, and computers all need to be in place before you start digitising.

Find the necessary floor space for your imaging infrastructure and buy/rent the necessary parts for the installation like cameras, lighting, tables, computers, software, storage, logo of your institution, colour bars and scales.

### ﻿7. Make your collection look its best for the photographer

**Figure 3.**
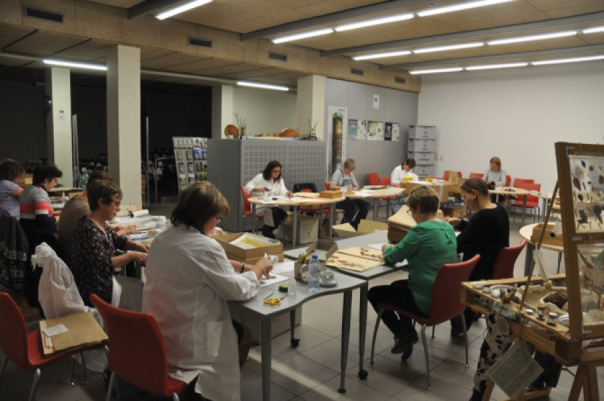
Joint restoration session of the herbarium team at Meise Botanic Garden.

In preparing your specimens for imaging, incorporate some pre-digitisation curation steps into your process like repairing and restoring specimens that may not be in optimal condition (Fig. 3; case study 1). This can be combined with adding a barcode to each sheet and marking specimens that don’t need to be photographed, for example because an image already exists of the specimen.

When opportune you can rearrange the sheets and update taxonomy at the same time. If the specimens need to be transported before imaging, appropriate packaging is needed (Giraud et al. 2019). Also think about pest treatment in the whole process, especially when the specimens are leaving the collection building (Giraud et al. 2019).

Remember to retrieve specimens loaned out to other institutes to make your collection as complete as possible before digitisation. Start this step well in advance because it may require considerable time to get your specimens back.

### ﻿8. Expect problems, particularly ones that you don’t expect

Equipment malfunctions and human mistakes are inevitable. You can do everything to avoid problems, but expect the unexpected. To avoid a problem turning into a disaster you will need a quality control process (Nieva de la Hidalga et al. 2020). This process should occur promptly after image capture, allowing for rephotographing of specimens before they are reshelved if issues arise. Some quality checks can be automated, including file size, embedded image metadata (i.e. Exif), barcode readability and conformity to standards, such as ISO 12234-2 for Tag Image File Format (TIFF). Other quality elements need human evaluation, such as focus, lighting and colour. All images cannot be inspected by a person, but a sub-sample can be inspected. If a problem is detected, then chances are high that the whole batch will have to be rejected, as quality issues tend to affect multiple images on production lines. Once the decision has been made to reject a batch there must be a mechanism to escalate the issue, correct the problem and clean the workflow of the rejected files.

Checking the quality of transcribed label data is another crucial step in the process. You will need a clear and detailed transcription protocol where your quality control process is set out (case study 2). Where possible, the use of lookup tables will greatly improve the quality of the transcribed data. Direct data quality control on a subset of the transcribed labels will help to reduce transcription errors (https://dissco.github.io/Digitisation/PreDigCuration/PDCaseStudies.html#quality-control-procedure-of-meise-botanic-garden-for-the-mass-digitisation-project-doe). This is particularly important at the beginning of the transcription process to weed out common errors and misunderstandings. Once the transcription process is complete, the data cleaning phase will start. Allow sufficient time for this task, as it often requires more time than initially anticipated. Tools like Open Refine (https://openrefine.org/) can be used for data cleaning. The process is often iterative, as corrected data in one field can often be used to fix data in another field.

### ﻿9. Make your data visible - make a big deal of it

**Figure 4.**
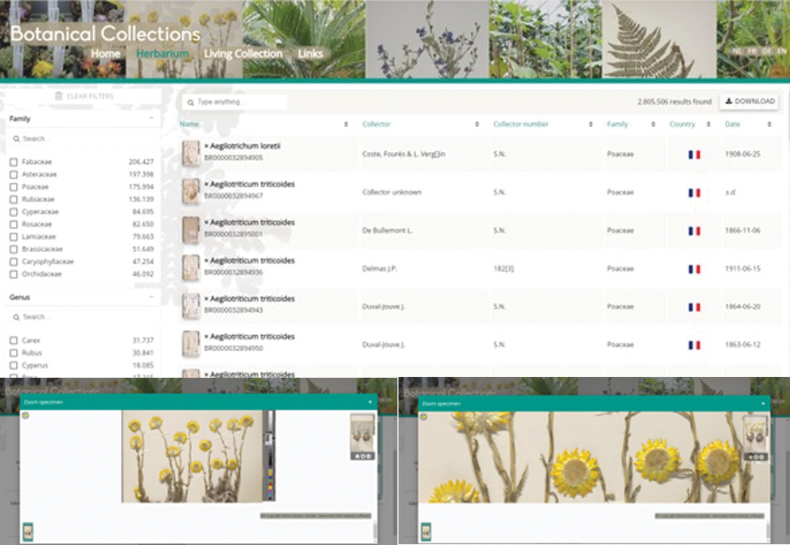
Screenshots of the virtual herbarium platform of Meise Botanic Garden (https://www.botanicalcollections.be), showing the start page and a detail of a specimen of *Helichrysumaureum*.

Digitisation of a herbarium is pointless if the data are not made publicly accessible. Don’t be shy; make sure everyone knows what you have achieved. Your herbarium is coming of age and it should be celebrated. To ensure that everyone can find and use your digitised herbarium, images and their metadata need to conform to the FAIR principles (Wilkinson et al. 2016). Institutions generally opt to create an online portal to their collection where they can showcase their specimens (Fig. 4). Make sure you are aware of the state of the art for such portals and how your specimens can be cited (Güntsch et al. 2017, case study 3). There are also other outlets for your images that will extend the reach of your collection to different communities. JSTOR Global Plants specialises on nomenclatural type and historical specimens, but is behind a paywall, while publishing on Wikimedia Commons allows your specimens to illustrate species on Wikipedia. As long as you use suitable licences, like CC BY or CC0, for your images, Wikipedians can use them freely. Your data should also be published to the Global Biodiversity Information Facility (https://www.gbif.org/publishing-data). A great advantage of this is that your dataset will be citable with a Digital Object Identifier (DOI). GBIF keeps track of publications citing their DOIs so you too can see who is using your data and why (https://www.gbif.org/literature-tracking).

Develop a Data Management Plan that outlines a strategy for handling, storing, and sharing of data (Dillen et al. 2024). Such a Data Management Plan aids in establishing clear guidelines for both internal staff and external users, specifying the terms governing data usage (including licensing) and storage.

### ﻿10. Save your data for the future

Backing up your data in a long-term offsite archive is an essential insurance against the loss of data and images. Depositing your images in that sort of cold storage should be part of the daily workflow of the digitisation pipeline. Think carefully about the formats of data too. Long-term storage formats, such as archive quality TIFF images, are not as suitable for day-to-day use as high quality jpg images. Storage will be an ongoing cost beyond the digitisation project and the institution will have to be able to sustain these costs (case study 4).

## ﻿Conclusions

Now that you have read our ten lessons, you should be ready to start your own mass digitisation project. Be well prepared, divide the work into manageable small tasks. When creating workflows, protocols, specifications and tenders, be as specific and detailed as possible, it will save you a lot of worries in the future.

When the mass digitisation process is complete you will reap the benefits: your collections will be more visible and used, digitisation will lead to even more projects and new types of research.

The daily management of the collection will also change, becoming more labour intensive: all incoming material needs to be digitised before entering the collection, errors will be more noticeable and need to be fixed, and the rearrangement of the collection needs to match the data in the collection management system. You not only have a physical collection to maintain but a digital one too!

## ﻿Case Study 1: Pre-digitisation curation

Our first and second mass digitisation projects employed different pre-digitisation approaches, because we had learned from the first project and adapted the workflow for some processes in the second project.

The first adjustment occurred in the preparation of herbarium sheets.

In the first project, 15 herbarium technicians worked 3 hours daily in the collection to make it ready for digitisation. Scattered throughout the collection, they meticulously checked every herbarium specimen, removed cellophane, plastic bags and paperclips, transferred loose material to envelopes, and added a barcode to each sheet. Sheets with multiple collections or specimens kept completely in envelopes were extracted from the collection to be digitised in-house. They also marked the specimens that were already digitised in the past, as well as sheets containing printed literature and pictures so the external digitisation company would recognise these as specimens not to digitise. A detailed workflow can be found on the following website: https://dissco.github.io/HerbariumSheets/MeiseBGHerbariumSheets.html#workflow.

It took us 1.5 years to finish the preparatory phase. This work came on top of the technicians’ regular work so some tasks needed to be postponed, such as processing incoming material and issuing loans. The labour-intensive nature of this process led to waning enthusiasm among staff.

Consequently, the approach for the second mass digitisation project underwent considerable revision. We decided to outsource the barcoding to an external company, a task which could be done at the conveyor belt when the specimens were laid out for imaging. Restoration efforts were drastically reduced, limited only to new arrivals, which were prepared on a weekly basis. During these weekly sessions, staff gathered in a dedicated room to check and restore specimens, having a little teambuilding moment at the same time while enjoying refreshments. This revised approach proved more sustainable and enjoyable, with preparation sessions now continued bi-weekly.

In the herbarium, the only task undertaken was tagging previously digitised specimens from past projects. Specimens in envelopes were tagged during transcription of the labels by the external company.

## ﻿Case Study 2: Label transcription

Numerous meetings were convened to determine the approach of label transcription and the specific fields to be transcribed. The outcome was to have multiple approaches depending on the collections. In general, we aimed to transcribe all key information fields essential for most scientific purposes, as agreed in de MIDS standards (Minimum Information about a Digital Specimen) (Hardisty et al. 2023). This resulted in a MIDS-2 level for most of the specimens.

At the start of our first project we opted to enter minimal data directly into our collection management system, BGBase. The fields that were transcribed in house by our technicians and volunteers included barcode, filing name, collector, collector number and country. For the central African specimens (specimens from DR Congo, Rwanda and Burundi) the phytoregion was also transcribed. This decision stemmed from budgetary constraints, as we were uncertain about funding for outsourcing transcription. For the remaining part of the African collection, we decided to outsource the transcription of the following fields: filing name, barcode, collector, collector number, country as given, country code, phytoregion, collection date, locality, altitude, altitude unit and coordinates as given. Label transcription was done based on the images by Alembo (https://alembo.nl/), a subcontractor of Picturae (https://picturae.com). A protocol was established by Picturae, Alembo and BR, and lookup lists were foreseen by BR for filing names, collectors, phytoregions (only for the first mass digitisation project) and countries. After multiple quality control steps (by Alembo, Picturae and Meise Botanic Garden), data was integrated in our collection management system.

For the Belgian herbarium, we created a multilingual crowdsourcing platform, DoeDat (www.DoeDat.be), where volunteers transcribed label information. We posted projects of around 2000 specimens per project so that citizens could help transcribe label information. We asked the public to transcribe all available label information on each specimen. We had foreseen a template with all possible fields and the necessary tutorials. DoeDat continues to assist with other collections. After finalisation of a DoeDat project, data is exported, cleaned and imported in our collection management system.

In the second mass digitisation project, the transcription of all specimens was outsourced to Alembo and Picturae. The same approach was used as in the first mass digitisation project, the same fields were requested in addition to collection name, curation notes with indications of multiple collections or envelope storage. These extra fields were added because of the different approach in pre-digitisation curation (see case study 1). The collection name field was added because the general herbarium contains much more different and older specimens, and thus handwritings that are more difficult to read than the African herbarium. This way we could easier group the specimens and facilitate the data cleaning afterwards.

The prospect of using Artificial Intelligence was not considered back in 2015, when we started the mass digitisation project. Although OCR was discussed, the high experimental cost at that time seemed to outweigh its potential benefits especially for handwritten labels. The use of AI for label transcription has since evolved considerably and should thus be considered in future digitisation projects.

All label information is made accessible on our virtual herbarium www.botanicalcollections.be.

## ﻿Case study 3: Development of the virtual herbarium portal and licensing

Given that our collection management system, BG-Base, lacks direct website integration capability, we opted to develop our own virtual herbarium portal www.botanicalcollections.be with assistance from an external IT consultant. We continue to rely on external development for new features and maintenance, while an internal staff member is responsible for communication and coordination between the Garden and the developer, as well as for minor changes and fixes. This approach, though resource-intensive due to the rapid evolution of online software technology, proves more cost-effective than hiring and retaining an internal developer with all the required expertise and experience.

On our data portal, users can search for specimens using the following fields: barcode, family, genus, species name, imaging status, type specimen, country, collector, collector number, collection year, kind of specimen and collection. A general search box that searches through a combined set of indexed data elements is also available. The interface can be set in all official Belgian languages (Dutch, French and German) as well as English.

Specimens on our data portal all have a unique stable identifier, following the CETAF persistent identifier specification, and this way the data is also available in machine-readable RDF format (Güntsch et al. 2017). There are also links to IPNI, Tropicos and BHL. The portal holds data from other Belgian herbaria as well, such as sub-collections of Ghent University (GENT) and the Université Libre de Bruxelles (BRLU). The domain name and site description were kept generic so as to cover the scope of making data available for any Belgian botanical collection. As digitisation of collections in Belgium continues, we expect more data from other collections to flow in.

It is possible to download data from our website in bulk for free, but a valid e-mail address is required for notification as such downloads are produced asynchronically. Data exported this way makes use of the Darwin Core data standard. Images can be downloaded through the interface at high resolution, but we do not offer an easy method to acquire them in bulk. This is a necessary evil to make it more difficult for web-scraping bots to overload our servers.

The website is hosted using the App Engine of Google Cloud services, to facilitate deployment of new features and make use of flexible hardware resources. Images are served from lossless JPEG2000 files through the Internet Imaging Protocol (IIP), to make them available for scientists with no reduction in quality or resolution. Given the large storage requirements for these files (> 200TB) and the associated high costs of actively storing them in the cloud, they are made available from servers hosted locally in the garden. Images are also available through a IIIF server that is similarly hosted at the Garden.

We make regular exports from our CMS to keep the data of the website up to date. We also host our own local IPT (Integrated Publishing Toolkit) instance to serve our specimen data to the Global Biodiversity Information Facility (GBIF), an international biodiversity data aggregator. The Botanical Collections data portal serves as the primary method to make our digitised collections available to the public. For GBIF, we apply an additional layer of quality control to ensure the associated data of specimens is as scientifically accurate as possible.

Data of our specimens is licensed under a CC BY licence and our images under a CC BY-SA licence (Dillen et al. 2024). Open data remains a contentious topic among the different users and providers to a herbarium collection. Development of the Botanical Collections portal was preceded by a stakeholder analysis (Vissers et al. 2017), from which a compromise vision for the portal’s access policies was distilled. Our data management plan has evolved over the years and all data is now available under a Creative Commons licence. Discussions on licenseless (Public Domain) publication of data continue and we have for instance published a minimal set of metadata this way to the Europeana platform.

## ﻿Case study 4: Image storage

In today’s digital age, the management of vast amounts of data, including images, is a crucial aspect. At Meise Botanic Garden, we employ a meticulous strategy for image storage, prioritising quality, security, and accessibility.

Our process for accepting and checking images is key to our storage system. Before archiving, each image goes through strict screening to meet our standards. Automated checks verify filename, file size, structure, resolution, and cropping, while visual inspections ensure quality and reliability (Nieva de la Hidalga et al. 2020).

After passing quality control, images are stored on two archive servers with capacities of 90TB and 112TB. Each server is backed up at a second location within the garden. We keep JPEG (jpg) and JPEG 2000 (jp2) versions locally for quick access, while the original TIFF files are stored with an external partner, Meemoo (https://meemoo.be). Meemoo employs cold storage on tape drives across three different locations, ensuring redundancy and long-term preservation. We use the TIFF format for long-term archiving due to its reliability and longevity. TIFF files provide a robust container for image data, safeguarding against format obsolescence and ensuring compatibility with future systems.

In our system, JPEG files serve as the go-to option for quick views on our portal, providing users with rapid access to image content. Conversely, JPEG 2000 files are instrumental in tiling via IIP MooViewer, enabling efficient and customizable image display across various platforms.

To facilitate access to archived images, we’ve implemented a reverse-proxy system coupled with an online webservice. This setup allows for quick queries, enabling our application to pinpoint the exact archiving server housing the required image. A Redis database in the background ensures rapid retrieval of image paths, enhancing efficiency.

## ﻿Additional information

### Author ORCIDs

Sofie De Smedt https://orcid.org/0000-0001-7690-0468

Ann Bogaerts https://orcid.org/0000-0003-3435-2605

Mathias Dillen https://orcid.org/0000-0002-3973-1252

Henry Engledow https://orcid.org/0000-0002-0779-8006

Frederik Leliaert https://orcid.org/0000-0002-4627-7318

Quentin Groom https://orcid.org/0000-0002-0596-5376

### Data availability

All of the data that support the findings of this study are available in the main text.
